# Blood leukocyte-based clusters in patients with traumatic brain injury

**DOI:** 10.3389/fimmu.2024.1504668

**Published:** 2025-01-09

**Authors:** Ruoran Wang, Jianguo Xu, Min He

**Affiliations:** ^1^ Department of Neurosurgery, West China Hospital, Sichuan University, Chengdu, Sichuan, China; ^2^ Department of Critical Care Medicine, West China Hospital, Sichuan University, Chengdu, Sichuan, China

**Keywords:** traumatic brain injury, leukocytes, clusters, phenotypes, K-means

## Abstract

**Background:**

Leukocytes play an important role in inflammatory response after a traumatic brain injury (TBI). We designed this study to identify TBI phenotypes by clustering blood levels of various leukocytes.

**Methods:**

TBI patients from the Medical Information Mart for Intensive Care-III (MIMIC-III) database were included. Blood levels of neutrophils, lymphocytes, monocytes, basophils, and eosinophils were collected by analyzing the first blood sample within 24 h since admission. Overall, TBI patients were divided into clusters following the K-means clustering method using blood levels of five types of leukocytes. The correlation between identified clusters and mortality was tested by univariate and multivariate logistic regression analyses. The Kaplan–Meier method was used to verify the survival difference between identified TBI clusters.

**Results:**

A total of 172 (cluster 1), 791 (cluster 2), and 636 (cluster 3) TBI patients were divided into three clusters with the following percentages, 10.8%, 49.5%, and 39.8%, respectively. Cluster 1 had the lowest Glasgow Coma Scale (GCS) and the highest Injury Severity Score (ISS) while cluster 2 had the highest GCS and the lowest ISS. The mortality rates of the three clusters were 25.6%, 13.3%, and 18.1%, respectively. The multivariate logistic regression indicated that cluster 1 had a higher mortality risk (OR = 2.211, p = 0.003) than cluster 2, while cluster 3 did not show a significantly higher mortality risk than cluster 2 (OR = 1.285, p = 0.163). Kapan–Meier analysis showed that cluster 1 had shorter survival than cluster 2 and cluster 3.

**Conclusion:**

Three TBI phenotypes with different inflammatory statuses and mortality rates were identified based on blood levels of leukocytes. This classification is helpful for physicians to evaluate the prognosis of TBI patients.

## 1 Introduction

Occurring widely with an estimated incidence of 69 million each year globally, traumatic brain injury (TBI) brings huge burdens to families of patients and to social economy (1). The mortality of TBI patients is high, especially severe TBI, with the mortality rate ranging from 23.0%–38.8% (2, 3). Accurate and convenient risk stratification of admitted TBI patients in the early phase is helpful for clinicians in making personalized treatments. The conventional tool of risk stratification for TBI is the Glasgow Coma Scale (GCS), which is evaluated based on clinical symptoms. However, single GCS could not comprehensively reflect the severity and progression of TBI patients due to the complex pathophysiological process of TBI and would be influenced by intubation and status of sedation. Identifying TBI phenotypes with different risks of clinical outcomes using laboratory biomarkers may make up for the insufficiency of GCS and guide clinicians in making specific treatment options based on pathophysiological processes.

Previous studies have explored phenotypes of some critically ill patients with acute kidney injury, sepsis, or acute respiratory distress syndrome using clustering methods (4–7). Composed of neutrophils, lymphocytes, monocytes, basophils, and eosinophils, leukocytes are recruited to the injured brain tissue and activated to participate in neuroinflammation after TBI (8–13). Previous studies have used the value of a single type of leukocytes or the neutrophil-to-lymphocyte ratio to reflect the inflammatory status and predict the prognosis of TBI (14–19). There is no study yet using blood values of all types of leukocytes to evaluate the prognosis of TBI. We designed this study to identify TBI phenotypes based on blood values of leukocytes using the K-means clustering method.

## 2 Materials and methods

### 2.1 Patients

The data for this study were extracted from the Medical Information Mart for Intensive Care-III (MIMIC-III) database produced by the Beth Israel Deaconess Medical Center (BIDMC). This database was approved by the institutional review boards of the Massachusetts Institute of Technology (MIT) and BIDMC. All patients included in this database were de-identified and anonymized for privacy protection. Written informed consent was waived due to the nature of the database study. The study design was approved by the review board of West China Hospital (2021-1598).

**Figure 1:**
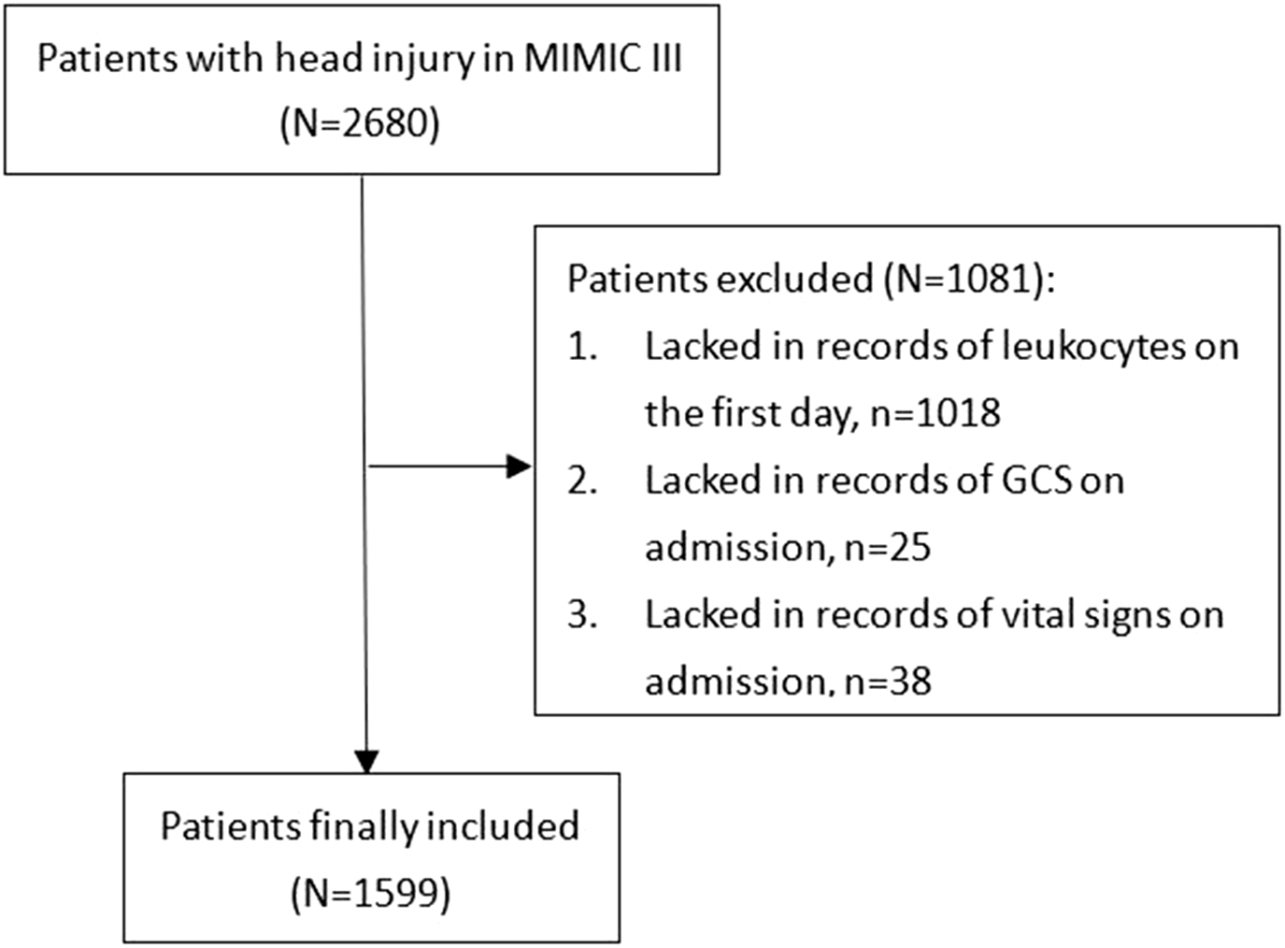
Flowchart of the patients’ inclusion.

The MIMIC-III is a free, public database of collected clinical records of critically ill patients who received treatments in intensive care units of the BIDMC (Boston, MA) between 2001 and 2012. The MIMIC-III database was designed and produced by MIT (Cambridge, MA) and received ethical approval from the institutional review boards of MIT and BIDMC, respectively. The diagnosis of TBI was identified according to ICD-9 codes: 80000-80199; 80300-80499; and 8500-85419. Overall, from 2,680 TBI patients, some were excluded due to the following standards: (1) lacked records of leukocytes on the first day (n = 1,018); (2) lacked records of GCS on admission (n = 25); and (3) lacked records of vital signs on admission (n = 38) (**Figure 1**). A total of 1,599 TBI patients were finally included in the study.

### 2.2 Data collection

Baseline characteristics including age, gender, blood pressure, SpO_2_, and comorbidities (diabetes, hypertension, hyperlipidemia, coronary heart disease, and cancer) were recorded. Disease severity was evaluated by including Glasgow Coma Scale (GCS), Injury Severity Score (ISS), and Sequential Organ Failure Assessment (SOFA). The values of blood biochemical and blood routine examination were obtained by analyzing the first blood sample within 24 h since admission. The number of leukocytes including neutrophils, lymphocytes, monocytes, basophils, and eosinophils was part of the blood routine examination.

### 2.3 Statistical analysis

Overall, TBI patients were divided into clusters by the unsupervised K-means clustering method using values of five kinds of leukocytes (neutrophils, lymphocytes, monocytes, basophils, and eosinophils). The K-means clustering is an iterative algorithm that involves pre-dividing data into K groups, randomly selecting K objects as the initial clustering centers, and then calculating the distance between each object and each seed clustering center. Each object is assigned to the nearest cluster center. The cluster centers and the objects assigned to them represent a cluster. The optimal number of clusters was determined according to the gap statistic criterion. The differences in baseline characteristics and clinical outcomes among clusters were analyzed by Kruskal–Wallis test and Pearson’s χ^2^ test. Additionally, inflammatory markers including neutrophil-to-lymphocyte ratio (NLR), monocyte-to-lymphocyte ratio (MLR), platelet-to-lymphocyte ratio (PLR), neutrophil-to-monocyte ratio (NMR), and systemic inflammation index (SII = platelet × neutrophil/lymphocyte) were compared between clusters. The correlation between TBI clusters and mortality was confirmed by univariate and multivariate logistic regression analyses. The Kaplan–Meier method was used to verify the survival difference among identified TBI clusters.

A two-sided p value < 0.05 was considered statistically significant. The R software (version 3.6.1; R Foundation) was used for statistical analyses.

## 3 Results

### 3.1 Comparison of characteristics among identified TBI clusters

**Figure 2:**
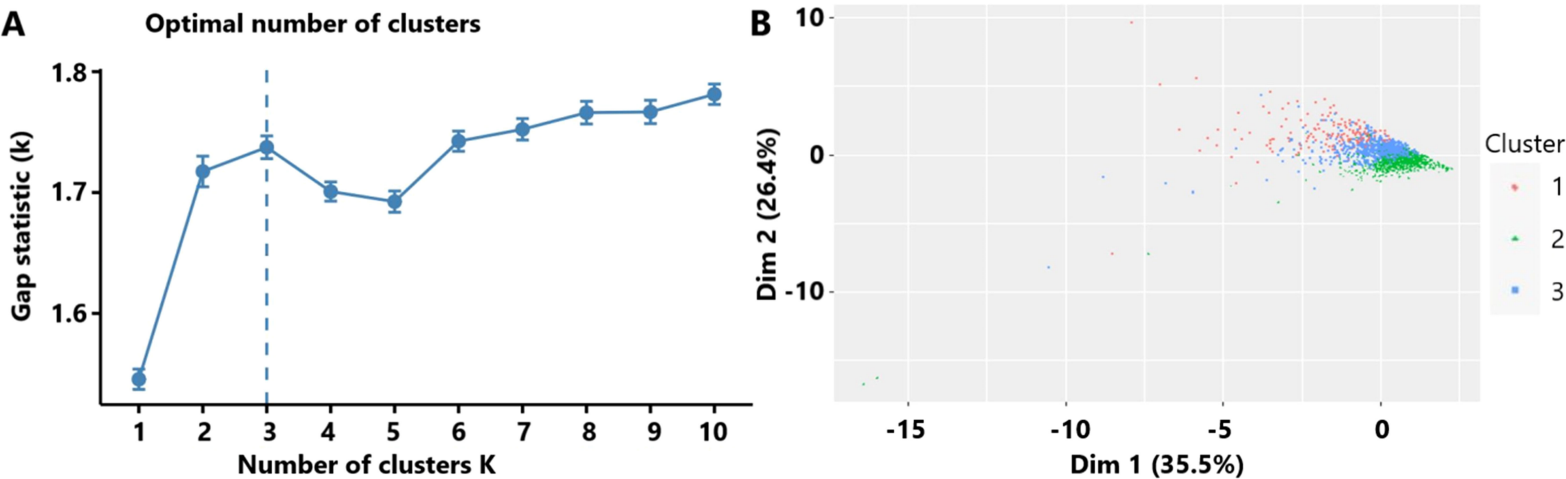
**(A)** The optimal number of clusters as determined by the gap statistic criterion. **(B)** Clusters’ plot. These two components explain the 61.9% point variability.

**Figure 3:**
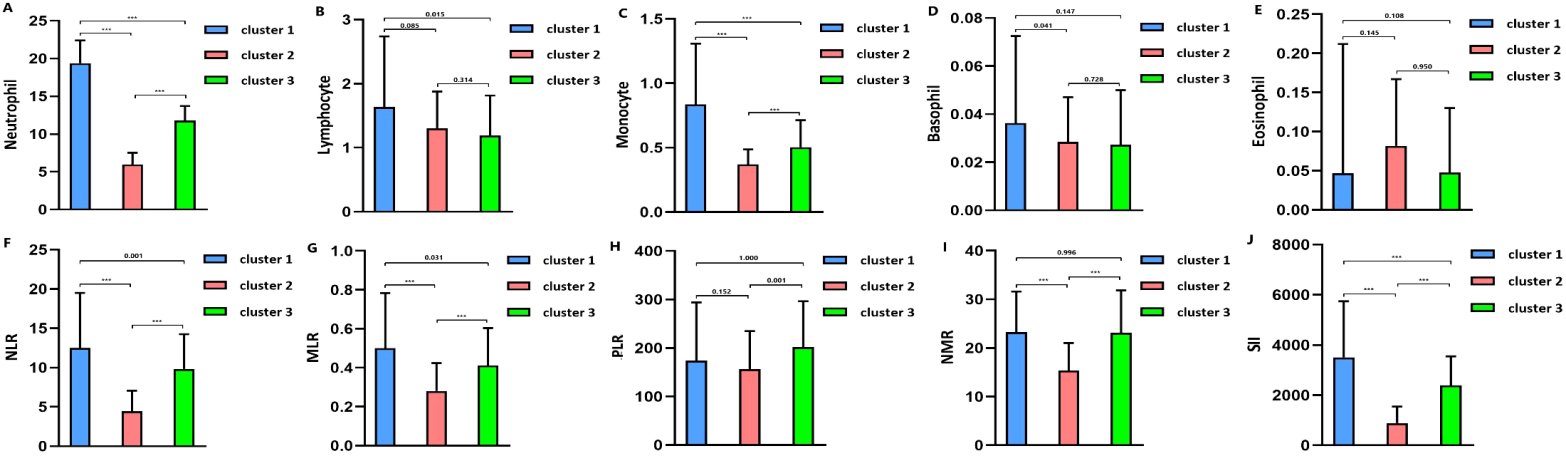
**(A)** Blood neutrophil level in clusters. **(B)** Blood lymphocyte level in clusters. **(C)**. Blood monocyte level in clusters. **(D)** Blood basophil level in clusters. **(E)** Blood eosinophil level in clusters. **(F)** Neutrophil-to-lymphocyte ratio (NLR) level in clusters. **(G)** Monocyte-to-lymphocyte (MLR) level in clusters. **(H)** Platelet-to-lymphocyte (PLR) level in clusters. **(I)** Neutrophil-to-monocyte (NMR) level in clusters. **(J)** Systemic inflammation index (SII) level in clusters. *** means p value<0.001.

**Figure 4:**
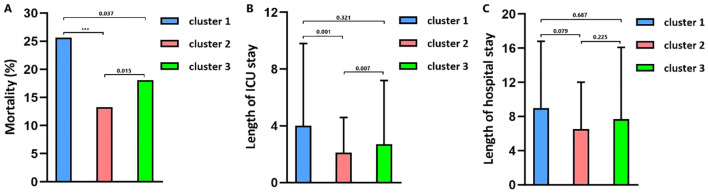
**(A)** The 30-day mortality in clusters. **(B)** Length of ICU stay in clusters. **(C)** Length of hospital stay in clusters.

The optimal number of TBI clusters determined by the gap statistic criterion was 3 (**Figure 2A**). Two components of the cluster plots explained the 61.9% variability of the point (**Figure 2B**). A total of 172, 791, and 636 patients were divided into cluster 1, cluster 2, and cluster 3, respectively, with percentages of 10.8%, 49.5%, and 39.8% (**Table 1**). Age (p < 0.001), incidence of comorbidities including diabetes (p = 0.008), hypertension (p < 0.001), hyperlipidemia (p = 0.004), coronary heart disease (p = 0.019), and cancer (p < 0.001) showed different distributions among the three clusters. Cluster 1 had the lowest GCS and the highest ISS while cluster 2 had the highest GCS and the lowest ISS. The SOFA score did not differ among the three clusters (p = 0.256). Cluster 1 had the highest value of WBC, neutrophil, lymphocyte, monocyte, platelet, RBC, hemoglobin, and glucose (**Figure 3**). Cluster 2 had the lowest value of WBC, neutrophil, monocyte, platelet, RBC, hemoglobin, and glucose. Regarding inflammatory markers, cluster 1 showed a higher inflammatory status reflected by higher levels of NLR, MLR, PLR, NMR, and SII. In contrast, cluster 2 showed a lower inflammatory status indicated by lower levels of NLR, MLR, PLR, NMR, and SII. Similarly, cluster 1 had a higher incidence of mechanical ventilation, 30-day mortality, and longer length of ICU stay and length of hospital stay (**Figure 4**). Cluster 2 had a lower incidence of mechanical ventilation, 30-day mortality, and shorter length of ICU stay and length of hospital stay.

**Table 1:** Baseline characteristics of included TBI patients.

Variables	Overall patients (n=1599)	Cluster 1 (n=172, 10.8%)	Cluster 2 (n=791, 49.5%)	Cluster 3 (n=636, 39.8%)	p
Age (years)	67.6 (47.2-82.3)	49.1 (28.0-73.9)	71.6 (54.0-83.2)	66.7 (43.4-82.2)	<0.001
Male gender (n, %)	959 (60.0 %)	108 (62.8%)	475 (60.1%)	376 (59.1%)	0.683
Comorbidities					
Diabetes (n, %)	271 (16.9%)	18 (10.5%)	154 (19.5%)	99 (15.6%)	0.008
Hypertension (n, %)	642 (40.2%)	45 (26.2%)	354 (44.8%)	243 (38.2%)	<0.001
Hyperlipidemia (n, %)	234 (14.6%)	16 (9.3%)	138 (17.4%)	80 (12.6%)	0.004
Coronary heart disease (n, %)	238 (14.9%)	14 (8.1%)	131 (16.6%)	93 (14.6%)	0.019
Cancer (n, %)	201 (12.6%)	12 (7.0 %)	133 (16.8%)	56 (8.8%)	<0.001
Systolic blood pressure (mmHg)	133 (117-148)	126 (112-144)	135 (120-150)	132 (116-147)	<0.001
Diastolic blood pressure (mmHg)	67 (56-78)	63 (56-75)	67 (57-79)	67 (55-78)	0.110
SpO_2_ (%)	99 (97-100)	100 (98-100)	98 (96-100)	99 (97-100)	<0.001
GCS	13 (7-15)	7 (5-12)	14 (9-15)	10 (6-15)	<0.001
ISS	16 (16-22)	22 (16-29)	16 (16-17)	16 (16-25)	<0.001
SOFA	3 (1-4)	3 (1-5)	3 (1-4)	3 (1-4)	0.256
Intracranial injury types					
EDH (n, %)	322 (20.1%)	54 (31.4%)	119 (15.0%)	149 (23.4%)	<0.001
SDH (n, %)	909 (56.8%)	86 (50.0%)	481 (60.8%)	342 (53.8%)	0.005
SAH (n, %)	589 (36.8%)	78 (45.3%)	259 (32.7%)	252 (39.6%)	0.001
Laboratory tests					
WBC (10^9/L)	11.30 (8.20-15.50)	23.30 (20.70-26.80)	8.20 (6.60-9.70)	14 (12.40-16.20)	<0.001
Neutrophil (10^9/L)	9.04 (6.05-12.72)	19.40 (17.80-22.40)	5.97 (4.53-7.53)	11.78 (10.50-13.72)	<0.001
Lymphocyte (10^9/L)	1.26 (0.85-1.91)	1.64 (1.03-2.72)	1.31 (0.85-1.88)	1.19 (0.82-1.81)	<0.001
Monocyte (10^9/L)	0.44 (0.32-0.64)	0.84 (0.62-1.31	0.37 (0.28-0.49)	0.50 (0.36-0.71)	<0.001
Basophil (10^9/L)	0.03 (0.01-0.05)	0.04 (0-0.07)	0.03 (0.01-0.05)	0.03 (0.01-0.05)	0.387
Eosinophil (10^9/L)	0.07 (0.02-0.16)	0.05 (0-0.19)	0.08 (0.03-0.17)	0.05 (0.02-0.13)	<0.001
Platelet (10^9/L)	230 (184-286)	287 (228-344)	212 (161-267)	237 (198-292)	<0.001
RBC (10^9/L)	4.12 (3.66-4.57)	4.38 (3.89-4.77)	4.05 (3.59-4.45)	4.17 (3.71-4.62)	<0.001
Hemoglobin (g/dL)	12.7 (11.2-14.1)	13.3 (11.9-14.7)	12.5 (11.1-13.8)	12.9 (11.4-14.2)	<0.001
Glucose (mg/dL)	131 (109-163)	152 (125-187)	118 (102-148)	142 (118-174)	<0.001
Prothrombin time (s)	13.1 (12.3-14.4)	13.4 (12.6-14.5)	13.2 (12.3-14.6)	13.0 (12.3-14.2)	0.128
Inflammatory markers					
NLR	6.92 (4.01-11.74)	12.50 (7.50-19.40)	4.42 (2.70-7.05)	9.84 (6.54-14.22)	<0.001
MLR	0.35 (0.23-0.55)	0.50 (0.36-0.77)	0.28 (0.19-0.42)	0.41 (0.29-0.60)	<0.001
PLR	174.89 (114.25-267.03)	174.95 (105.02-293.63)	156.78 (103.09-234.09)	202.55 (128.62-296.06)	<0.001
NMR	18.66 (13.21-27.42)	23.184 (15.60-31.52)	15.46 (11.00-21.30)	23.162 (16.60-31.82)	<0.001
SII	1597.50 (821.59-2856.91)	3521.05 (2157.55-5700.14)	887.66 (527.63-1537.54)	2400.64 (1497.13-3552.40)	<0.001
Platelet transfusion during the first day (n, %)	152 (9.5%)	10 (5.8%)	95 (12.0%)	47 (7.4%)	0.003
RBC transfusion during the first day (n, %)	105 (6.6%)	13 (7.6%)	50 (6.3%)	42 (6.6%)	0.837
Mechanical ventilation (n, %)	688 (43.0%)	107 (62.2%)	257 (32.5%)	324 (50.9%)	<0.001
30-day mortality (n, %)	264 (16.5%)	44 (25.6%)	105 (13.3%)	115 (18.1%)	<0.001
Length of ICU stay (days)	2.4 (1.2-6.0)	4.0 (2.0 -9.8)	2.1 (1.1-4.6)	2.7 (1.3-7.2)	<0.001
Length of hospital stay (days)	6.9 (3.8-14.4)	9.0 (4.5-16.8)	6.5 (3.7-12.0)	7.7 (3.6-16.1)	0.003

SpO_2_, pulse oxygen saturation; GCS, Glasgow Coma Scale; ISS, Injury Severity Score; SOFA, sequential organ failure assessment; EDH, epidural hematoma; SDH, subdural hematoma; SAH, subarachnoid hemorrhage; WBC, white blood cell; RBC, red blood cell; NLR, neutrophil to lymphocyte ratio; MLR, monocyte to lymphocyte ratio; PLR, platelet to lymphocyte ratio; NMR, neutrophil to monocyte ratio; SII, systemic immune inflammation index = platelet × neutrophil/lymphocyte.

### 3.2 Relationship between identified clusters and outcomes of TBI

**Figure 5:**
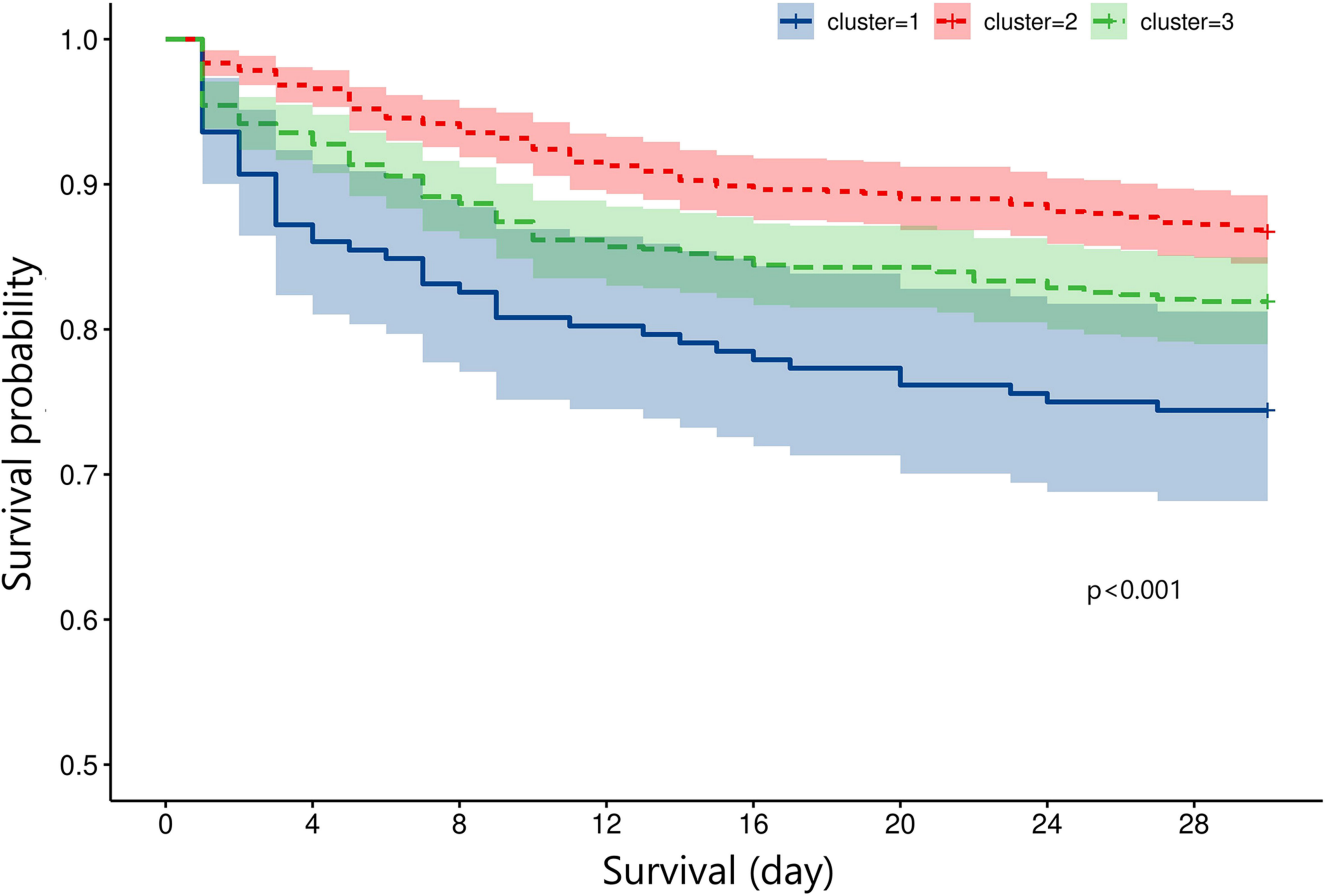
Survival curve of the three clusters by the Kaplan–Meier method.

The mortality rates of the three clusters were 25.6%, 13.3%, and 18.1%, respectively. Univariate regression showed that cluster 1 (OR = 2.246, p < 0.001) and cluster 3 (OR = 1.442, p = 0.003) had higher mortality risks than cluster 2 (**Table 2**). Additionally, age (p < 0.001), diabetes (p < 0.001), hypertension (p = 0.216), cancer (p = 0.005), diastolic blood pressure (p = 0.038), GCS (p < 0.001), ISS (p < 0.001), SOFA (p < 0.001), SAH (p = 0.040), platelet (p = 0.016), RBC (p < 0.001), hemoglobin (p < 0.001), glucose (p < 0.001), and prothrombin time (p = 0.006) were significantly associated with TBI mortality. After adjusting the confounding effects of those factors, multivariate logistic regression indicated that cluster 1 had a higher mortality risk (OR = 2.211, p = 0.003) than cluster 2 while cluster 3 did not show a significantly higher mortality risk than cluster 2 (OR = 1.285, p = 0.163). Kapan–Meier analysis found that cluster 1 (p < 0.001) had shorter survival than cluster 2 (p < 0.001) and cluster 3 (p = 0.024; **Figure 5**). Cluster 3 had shorter survival than cluster 2 (p = 0.009).

**Table 2:** Univariate and multivariate logistic regression analysis of risk factors for mortality in TBI patients.

	Unadjusted analysis			Adjusted analysis		
Variables	OR	95% CI	P value	OR	95% CI	P value
Age	1.027	1.020-1.035	<0.001	1.046	1.036-1.057	<0.001
Male gender	0.840	0.643-1.097	0.200			
Diabetes	1.790	1.304-2.458	<0.001	1.002	0.993-1.011	0.628
Hypertension	1.183	0.906-1.545	0.216			
Hyperlipidemia	1.205	0.842-1.725	0.307			
Coronary heart disease	1.137	0.793-1.631	0.483			
Cancer	1.661	1.162-2.374	0.005	1.288	0.859-1.931	0.220
Systolic blood pressure	0.999	0.993-1.004	0.610			
Diastolic blood pressure	0.992	0.984-1.000	0.038	1.569	1.028-2.397	0.037
SpO_2_ (%)	0.998	0.968-1.029	0.900			
GCS	0.857	0.831-0.883	<0.001	0.833	0.801-0.865	<0.001
ISS	1.032	1.017-1.047	<0.001	1.033	1.013-1.054	0.001
SOFA	1.273	1.210-1.338	<0.001	1.162	1.093-1.234	<0.001
EDH	1.112	0.805-1.535	0.519			
SDH	0.999	0.765-1.304	0.991			
SAH	1.325	1.013-1.734	0.040	1.527	1.114-2.095	0.009
Leukocytes clusters			<0.001			0.012
Cluster 2	1.000	Reference		1.000	Reference	
Cluster 1	2.246	1.506-3.348	<0.001	2.211	1.310-3.733	0.003
Cluster 3	1.442	1.081-1.923	0.003	1.285	0.903-1.827	0.163
Platelet	0.998	0.997-1.000	0.016	1.855	1.162-2.960	0.010
RBC	0.659	0.547-0.793	<0.001	0.782	0.669-0.915	0.002
Hemoglobin	0.837	0.788-0.890	<0.001	0.999	0.997-1.001	0.260
Glucose	1.009	1.006-1.011	<0.001	1.003	1.000-1.005	0.035
Prothrombin time	1.022	1.006-1.037	0.006	1.015	0.999-1.031	0.062

OR, odds ratio; CI, confidence interval; SpO_2_, pulse oxygen saturation; GCS, Glasgow Coma Scale; ISS, Injury Severity Score; SOFA, sequential organ failure assessment; EDH, epidural hematoma; SDH, subdural hematoma; SAH, subarachnoid hemorrhage; RBC, red blood cell.

## 4 Discussion

We found that the three TBI clusters had different inflammatory statuses through the clustering of peripheral leukocytes. Cluster 1 with a higher inflammatory status had a worse prognosis than cluster 2 with a lower inflammatory status. TBI could induce an increase in leukocytes soon after an initial injury. Neutrophils would be released from the bone marrow by stimulating cortisol and catecholamines after TBI (20). The activated microglia after TBI would then activate the endothelial cell and recruit the peripheral neutrophil to infiltrate the injured area by releasing inflammatory chemokines and cytokines (8, 9). Then, activated T cells and monocytes/macrophages would be recruited to the injured brain area and participate in the response of the adaptive immune system for the injured brain tissue (10–13). These cells would interactively activate and promote a secondary brain injury by aggravating neuroinflammation and immune response (21).

The relatively lower level of lymphocytes in the cluster and the higher level of lymphocytes in cluster 2 indicated that the peripheral immunosuppressive status might be related with a higher inflammatory status and worse outcome. Actually, previous studies found that TBI could cause the acute increase of cortisol levels both in serum and cerebrospinal fluid (22, 23). The increased plasma cortisol in the early phase after TBI would prevent the lymphocyte egress from the secondary lymphoid tissues, which may be an endogenous protective response to inhibit the excessive infiltration of T cells in the injured brain area, subsequently amplifying neuroinflammation (24). However, the peripheral immunosuppressive status is certainly associated with a higher risk of infection among extracranial organs.

Many previous studies used the value of a single type of leukocytes or neutrophil-to-lymphocyte ratio to evaluate the risk of poor prognosis of TBI (14–19), while not all types of leukocytes were included and analyzed to evaluate the prognosis risk, which was a limitation of these studies. We performed this study to identify inflammatory clusters of TBI patients with different mortality risks by comprehensively clustering multiple types of leukocytes, namely, neutrophils, lymphocytes, monocytes, basophils, and eosinophils. Three inflammatory clusters of TBI were identified and were consistent with their GCS. This classification is beneficial for physicians to stratify risks and make personalized treatment schedules. It may also be helpful to identify TBI subgroups benefiting from specific treatments targeted at inflammation.

Several limitations were unavoidable in this study. Firstly, a number of patients were excluded from this study mainly due to lack of records on leukocytes on the first day, causing a selection bias. The classification we identified should be further verified in other medical centers with more generalized TBI patients and a prospective design collecting levels of leukocytes at a fixed time period, as early as possible, after admission. Secondly, specific subgroups of lymphocytes were not analyzed including NK cells, B cells, cytotoxic T cells, and regulatory T cells due to lack of records from the MIMIC-III database. Thirdly, common inflammation markers such as C-reactive protein, interleukin-1, interlukin-6, TNF-α, and interferon-γ were not analyzed among the three clusters due to lack of records from MIMIC-III. Future studies could be performed to measure these markers among our identified clusters and evaluate the consistency between these markers and clusters with different inflammatory statuses. Fourthly, functional outcomes and recovery statuses were not recorded in MIMIC-III, so we were unable to analyze the relationship between the discovered three clusters and these outcomes. Future studies could be designed to verify the importance of the three clusters on functional outcomes and recovery statuses. Finally, agents with immunomodulatory effects or cytotoxicity were not particularly extracted and analyzed, which may limit the reliability of our classifications.

## 5 Conclusion

Three TBI clusters with different inflammatory statuses and prognoses were identified based on levels of blood leukocytes. This classification is beneficial for physicians in evaluating the prognosis and making personalized treatments for TBI patients.

## Data Availability

Publicly available datasets were analyzed in this study. This data can be found here: https://physionet.org/content/mimiciii/1.4/.
